# Boosting laser-ion acceleration with multi-picosecond pulses

**DOI:** 10.1038/srep42451

**Published:** 2017-02-13

**Authors:** A. Yogo, K. Mima, N. Iwata, S. Tosaki, A. Morace, Y. Arikawa, S. Fujioka, T. Johzaki, Y. Sentoku, H. Nishimura, A. Sagisaka, K. Matsuo, N. Kamitsukasa, S. Kojima, H. Nagatomo, M. Nakai, H. Shiraga, M. Murakami, S. Tokita, J. Kawanaka, N. Miyanaga, K. Yamanoi, T. Norimatsu, H. Sakagami, S. V. Bulanov, K. Kondo, H. Azechi

**Affiliations:** 1 Institute of Laser Engineering, Osaka University, Suita 565-0871, Osaka, Japan; 2 PRESTO, Japan Science and Technology Agency, Kawaguchi, Saitama 332-0012, Japan; 3 The Graduate School for the Creation of New Photon Industries, Hamamatsu, Shizuoka 431-1202, Japan; 4 Graduate School of Engineering, Hiroshima University, Higashi-Hiroshima 739-8511, Japan; 5 Kansai Photon Science Institute, National Institutes for Quantum and Radiological Science and Technology, Kizugawa 619-0215, Kyoto, Japan; 6 National Institute for Fusion Science, Gifu 509-5292, Japan

## Abstract

Using one of the world most powerful laser facility, we demonstrate for the first time that high-contrast multi-picosecond pulses are advantageous for proton acceleration. By extending the pulse duration from 1.5 to 6 ps with fixed laser intensity of 10^18^ W cm^−2^, the maximum proton energy is improved more than twice (from 13 to 33 MeV). At the same time, laser-energy conversion efficiency into the MeV protons is enhanced with an order of magnitude, achieving 5% for protons above 6 MeV with the 6 ps pulse duration. The proton energies observed are discussed using a plasma expansion model newly developed that takes the electron temperature evolution beyond the ponderomotive energy in the over picoseconds interaction into account. The present results are quite encouraging for realizing ion-driven fast ignition and novel ion beamlines.

The acceleration of energetic ions driven by relativistic-intensity (>10^18^ W cm^−2^) laser pulses^1^,^2^ is attracting large interest based on the prospects of realizing a novel source of intense ions for oncology^3^,^4^ or ion-driven fast ignition^5^,^6^. In the past, detailed investigations were performed with laser intensities on the order of 10^18^–10^20^ W cm^−2^, where relatively thick (~*μ*m) solid foils are used as laser-irradiation targets and the ion acceleration is well explained by the Target Normal Sheath Acceleration (TNSA) mechanism^7^,^8^,^9^,^10^. Nowadays, considerable interest is being paid to mono-energetic ion acceleration using radiation pressure acceleration (RPA)^11^,^12^,^13^, breakout afterburner (BOA)^14^,^15^, magnetic vortex acceleration (MVA)^16^,^17^,^18^,^19^,^20^ and collisionless shock acceleration (CSA)^21^,^22^. In the MVA and CSA schemes, laser pulses are injected into low density plasmas produced by gas targets or exploding thin foil. RPA and BOA require nanometer-thick solid targets satisfying the condition of relativistic transparency.

In the TNSA regime, ions on the target rear surface (opposite to the laser irradiated side) are accelerated by a charge separation field, which can be described by the expansion of the rear-side plasma into vacuum. Namely, the ions are accelerated by the electrostatic field generated by the electron pressure gradient^23^,^24^. The maximum ion energies found in several experiments in the TNSA regime have been reasonably scaled by the formula of Fuchs *et al*.^25^, based on a model^26^ describing the ion acceleration from a plasma front in the framework of one-dimensional (1D) isothermal expansion. They assumed the temperature of hot electrons to be constant at the *ponderomotive* energy^27^,


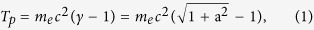


where *m*_*e*_ is the static mass of an electron, *c* is the speed of light in vacuum, *γ* is the Lorentz factor and **a** is the normalized vector potential of the laser defined by **a** = *e***A**/*m*_*e*_*c*^2^ with the elementary charge *e* and the vector potential of the electromagnetic wave **A**. The time-dependence of the hot-electron temperature has been introduced in several studies^28^,^29^,^30^, where the hot-electron temperature begins to decrease through the adiabatic expansion of the plasma after the energy supply from the incident laser is switched off. Nowadays, growth of electron temperature via nonlinear mechanism^31^,^32^,^33^,^34^,^35^ has been reported.

In recent experiments, the maximum ion energy^36^,^37^,^38^,^39^ and the monochromaticity^40^,^41^,^42^ have been enhanced by the use of high-contrast, *i.e*. low background light, femtosecond (fs) pulses interacting with the thin foil target. For a picosecond laser, however, the foil target plasma is expanded by the ps main pulse, even though the background light is markedly suppressed. Until now, there have been no systematic experimental investigations using high-contrast laser pulses with a duration longer than 1 picosecond (ps), except ref. 43.

From the viewpoint of realizing a novel ion source, the conversion efficiency (CE) of laser energy into the ions is also one of the most important issues. Ion fast ignition in laser fusion assisted by laser-driven ion beams requires 10 kJ energy deposited onto the fuel core with ~500-g cm^−3^ density^6^. Assuming 100 kJ as a technically manageable energy of the driving laser, the first milestone can be found for the CE of 10%.

In this study, it is demonstrated for the first time that a high-contrast laser pulse with kilojoule energy and multi-ps duration accelerates ions efficiently with 5% CE. We show that the electron temperature is enhanced beyond the ponderomotive energy when the pulse duration is set to be optimum. These electron heating processes are investigated by experiments and particle-in-cell (PIC) simulations. We also demonstrate that the enhanced electron heating makes the maximum ion energy much higher than that for conventional scaling with high CE drastically improved by the multi-ps laser pulse.

## Results

### Overview

Here we report ion acceleration by high-contrast ps pulses obtained from one of the most powerful laser facilities in the world: LFEX^44^,^45^ of Osaka University. LFEX simultaneously delivers four laser beams with a full width at half maximum (FWHM) pulse duration of 1.5 ps. The maximum laser energy in total is 1 kJ (250 J for the each laser beam) on the target. In order to realize a planar plasma expanding in quasi-one dimension, the laser pulses are weakly focused by an F/10 off-axis parabolic mirror (OAP) into a spot of 60 *μ*m (FWHM), corresponding to a peak intensity of *I* = 2.3 × 10^18^ W cm^−2^ for the each laser beam. The laser pulse is normally incident on 5-*μ*m-thick aluminum foil. The ions accelerated from the rear side of the target are observed by a Thomson-parabola (TP) ion spectrometer located in the normal direction of the target rear surface. The electrons generated from the plasma are also measured by an electron spectrometer (ESM) located at the target rear side. The angle between the TP and the ESM is 20.9°.

One of the most outstanding features of LFEX is that it provides ps pulses with contrast that compares favorably with that of fs pulses from table-top laser systems: 10^14^ at a time 10 ns before the main pulse and 10^9^ at a time 200 ps before the main pulse. The intensities of these backgrounds are lower than typical atomic field ionization thresholds. Another characteristic of LFEX is that it consists of four beams that can be changed independently of the arrival time at the target, by adjusting the laser path length upstream of the OAP. By setting intervals of 1.5 ps between the four pulses, for instance, we can obtain a duration-extended pulse of 6 ps (FWHM) as the longest case using the four pulses. Thereby, we can keep the rising edge of the extended pulse to be exactly the same as that of the single pulse. If the pulse duration is extended by adjusting the pulse compressor of the laser system, the rising edge would inevitably be modified into a more gradual shape.

### Pulse duration dependency

**Figure 1:**
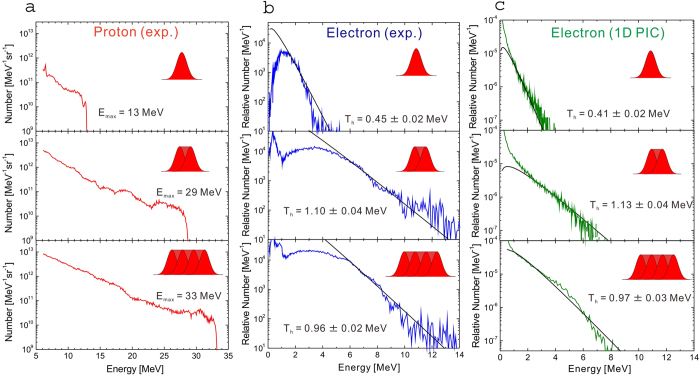
(**a**) Proton energies observed for different pulse durations, *t*_*L*_ = 1.5, 3 and 6 ps (FWHM), and a fixed laser intensity of *I* = 2.3 × 10^18^ W cm^−2^. (**b**) Electron energy spectra measured simultaneously with the protons, where the higher-energy part of the spectra exhibits a Maxwellian distribution 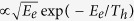 (black solid lines) as a function of the kinetic energy *E*_*e*_ and the hot-electron temperature *T*_*h*_ in units of energy. (**c**) Time-averaged electron energy spectra obtained with the 1D PIC simulation, the temperature slopes of which are in agreement with those of the experimentally observed spectra shown in (**b**).

Figure 1(a) shows the energy distribution of protons measured with the TP, when the pulse duration is varied with the “pulse-train” method mentioned above. Even though the laser intensity is fixed at *I* = 2.3 × 10^18^ W cm^−2^, the proton energy is enhanced from 13 MeV to 29 MeV when we expand the duration from *t*_*L*_ = 1.5 ps to 3 ps. In addition, the proton energy saturates around 30 MeV with a further long pulse duration (6 ps).

Another noteworthy result is found from the electron energy spectra analyzed simultaneously with the protons [Fig. 1(b)], where the electron temperature, derived from the higher-energy part of the spectra, grows to *T*_*h*_ = 1.10 MeV for the 3-ps duration and begins to decrease at 6 ps. Note that the drop from the Maxwellian line seen in the lower-energy part can be attributed to the fact that the sheath field on the plasma surface prevents lower energy electrons from escaping the plasma and reaching the ESM, which is located ~75 cm away from the target. The electron temperature observed here is higher by a factor of 5 than the ponderomotive energy evaluated from Eq. (1), *T*_*p*_ = 0.20 MeV, which depends on the laser intensity, while it has no dependence of the pulse duration. Here we used 
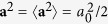
, where 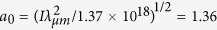 is the dimensionless amplitude of the laser with a wavelength of *λ*_*μm*_ = 1.053 *μ*m. High-temperature electrons beyond the ponderomotive energy were often observed in the interaction between foil targets and relatively lower-contrast laser pulses, where a preformed plasma at the target front side was the most probable source of the high-energy electrons. It was reported^46^ that such high-energy electrons from the preformed plasma do not play a major role in the ion acceleration from the target rear surface. In contrast, the main argument of this study is that the temperature of electrons grows as time and such evolving electron temperature plays important roles on ion acceleration in multi-ps interaction. The mechanism of temporal evolution of the electron temperature is revealed by 1D PIC simulations [Fig. 1(c)], demonstrating the growths of electron temperatures of 0.41, 1.13 and 0.97 MeV for 1.5-, 3- and 6-ps durations, respectively. Considering that the ESM used here has no time resolution, the PIC results are obtained by integrating the electron spectra over the entire time domain during the interaction. (For instance, we have acquired 68 electron spectra for the single-pulse case over a time domain of 3.2 ps). Under the current condition, the electron temperatures obtained with the 1D PIC simulation show quantitative agreement with those of ESM measurements, seen in Fig. 1.

In the next subsection, we discuss the evolution of the electron temperature depending on time using 1D PIC simulation over a long time domain reaching a maximum of 8 ps. We demonstrate that the enhanced proton energy is explained by the new approach of introducing the temporal evolution of electron heating into the theory of 1D plasma expansion.

### Temporal evolution of plasma temperature

**Figure 2:**
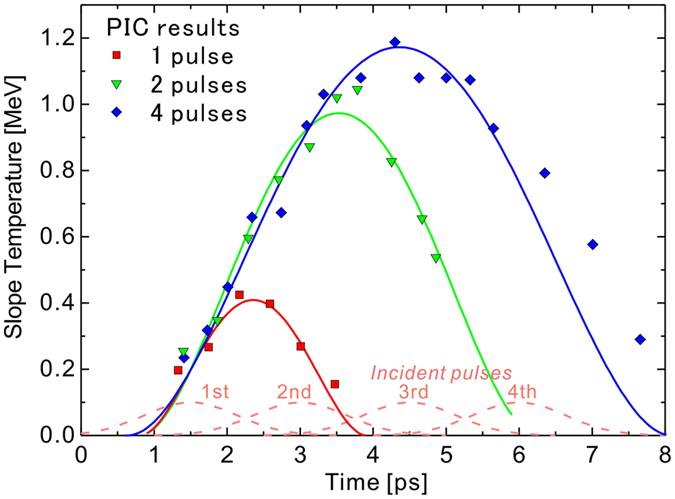
Time evolution of the temperature slope obtained with the PIC simulation for the incidence of a single pulse (*t*_*L*_ = 1.5 ps, red squares), 2-pulse train (*t*_*L*_ = 3 ps, green triangles) and 4-pulse train (*t*_*L*_ = 6 ps, blue diamonds). The temporal shape of the incident pulses used in the simulation are shown as dashed lines. The solid lines are determined by least squares fitting with Eq. (17); see the text.

We have performed 1D PIC simulations using the EPIC code^47^ to understand the time-dependent heating of plasma electrons depending on the laser pulse duration. Figure 2 shows the temporal variations of electron temperature for 1.5-, 3- and 6-ps pulse durations, corresponding to the incidence of a single pulse, two-pulse train and four-pulse train, respectively. Here, we assume a Gaussian profile for the temporal shape of each laser pulse and set a time of *t* = 1.5 ps for the arrival timing of the first intensity peak (*I* = 2.3 × 10^18^ W cm^−2^), as shown with dashed lines. The four pulses are separated by a time interval of 1.5 ps between the peaks, making the flattop-like shape of the pulse profile continue for *t* = 1.5–3 ps for the 2-pulse case and *t* = 1.5–6 ps for the 4-pulse case.

One can see at the first intensity peak (*t* = 1.5 ps) that the electron temperature reaches ~0.2 MeV, which is close to the ponderomotive energy *T*_*p*_ = 0.20 MeV, mentioned in the previous section. The temperature afterward keeps growing beyond the ponderomotive energy. In the cases of 1-pulse and 2-pulse incidence, the temperature reaches 0.4 MeV and 1 MeV, respectively, and begins to decrease as the laser intensity fades. The electron continues to be heated during the laser incidence, depending on time, and begins to cool down adiabatically^28^,^29^,^30^ when the laser is switched off. However, in the case of 4-pulse incidence, the temperature begins to decrease around *t* = 4.5 ps even though the flattop-like laser pulse still remains. This result is beyond the framework of the adiabatic cooling process. We can explain it according to the following scenario: For a too long duration (*t* = 4.5 ps, here), the cooling effect, attributed to plasma expansion, becomes so strong that it dominates over the effect of temporally evolution of electron heating during the laser incidence, resulting in a decrease in the electron temperature. This fact indicates that there is the optimum pulse duration for the electron heating to maximize the energy of accelerated ions.

**Figure 3:**
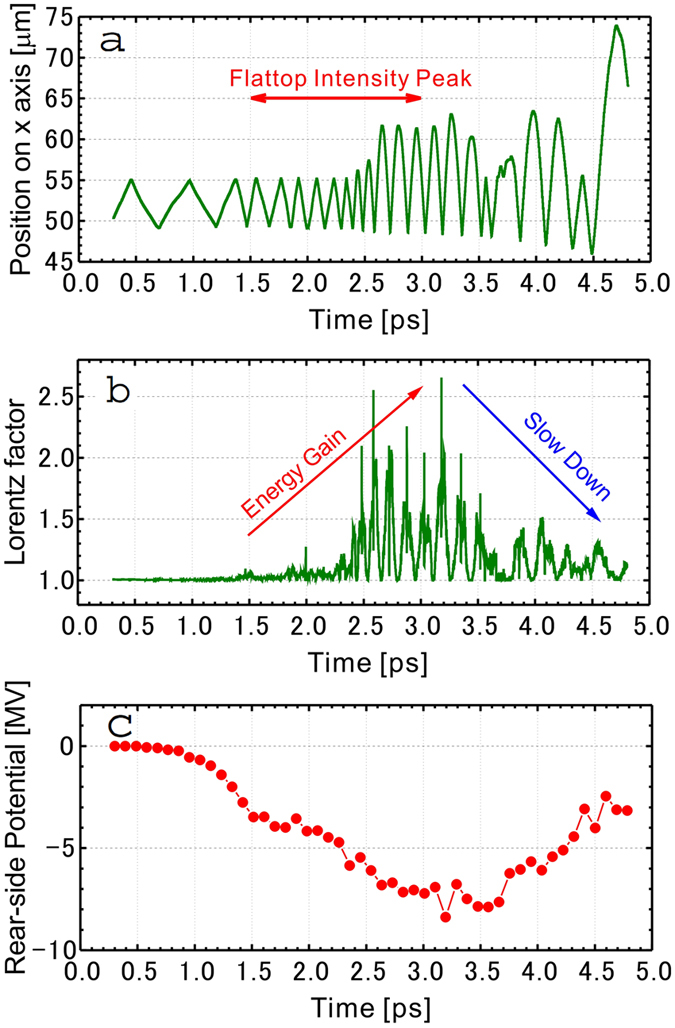
(**a**) Trace of typical electron trajectory in the 1D PIC simulation. The target foil is initially at the position *x* = 50–55 *μ*m and the laser (2-pulse train) is incident on the surface at *x* = 50 *μ*m. (**b**) Time evolution of the Lorentz factor of the electron shown in (**a**). (**c**) The temporal evolution of the potential generated on the rear side.

The driving mechanism underlying the electron heating is found in the hot electron trajectory tracked in the 1D PIC calculation. Figure 3(a) shows the trace of one electron motion in the laser propagation direction (positive direction on the *x* axis), where the target foil is initially located at *x* = 50–55 *μ*m. The incident laser is a 2-pulse train (3 ps on FWHM) and the time on the horizontal axis here corresponds to that of Fig. 2. The electron is accelerated forward from the front surface and pulled back by the potential generated on the target rear side. Then the electron is again pushed forward and continues recirculating around the target plasma. When the intensity reached the flattop peak (*t* > 1.5 ps), the electron recirculation frequency increases and the amplitude of the trajectory is jumping up. At the same time, the kinetic energy of the electron increases, as shown in Fig. 3(b). When the laser intensity is attenuated (*t* > 3 ps), the electrons slow down because of the adiabatic cooling accompanying the plasma expansion and the electron recirculation becomes less frequent.

In addition, we find that the electrostatic potential on the target rear side correlates with the electron motion discussed above. In Fig. 3(c), the rear-side potential reaches −9 MV around the endpoint of the flattop peak (3 ps). This fact indicates that even though the electrons are heated beyond the ponderomotive energy, they are reflected several times by the rear potential and continue to be heated. The growth of temperature generates a positive feedback to improve the rear-side potential. This is the reason why the electron temperature continues to increase until the flattop intensity peak ends.

In this study, the electrons are circulating around relatively thick region of the plasma, ~10 *μ*m in Fig. 3(a), resulting in a time period in the order of sub-ps. Hence, the heating mechanism discussed above is a characteristic phenomenon seen with multi-ps laser pulses, quite different from previous studies^48^,^49^,^50^ assuming fs pulses, which is shorter than the period of the electron recirculation seen in this study.

### Ion acceleration model

In the previous paragraph we clarified the characteristic mechanism of electron heating, that well explains the plasma temperature observed in the experiment. Here, we attempt to construct an ion acceleration model involving the temporally-evolved electron heating, based on the framework of 1D plasma expansion into vacuum^23^,^24^. We assume a one-fluid model with cold ions and hot electrons with a time-dependent temperature *T(t*) following the Boltzmann distribution,


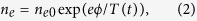


in a self-consistent potential *ϕ*. At *t* = 0, the plasma is assumed to occupy the region *x* < 0 and the ion density is *n*_*i*_ = *n*_*i*0_ for *x* < 0 and *n*_*i*_ = 0 for *x* > 0. The potential *ϕ* is given according to the Poisson equation,


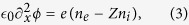


where *Z* is the ion charge number (*Z* = 1 for protons). The electric field at the border *x* = 0 is written as


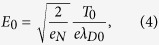


where *e*_*N*_ is Napier’s constant and 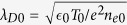 is the initial value of the Debye length with the ponderomotive scale temperature *T*_0_. The ion expansion into a vacuum is described by the hydrodynamic equations,


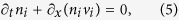



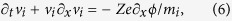


where *v*_*i*_ and *m*_*i*_ are the velocity and mass of the ions, respectively. We assume quasi-neutrality *n*_*e*_ = *Zn*_*i*_ in the regions where the spatial scale of the plasma is sufficiently larger than the Debye length *λ*_*D*0_. In this work, we introduce a new self-similar variable depending on time,


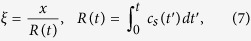


where 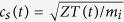 is the acoustic velocity of ions depending on time. Then, the solutions of the potential *ϕ* and electric field *E* are obtained in the forms


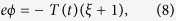



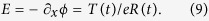


Attributed to the breaking of quasi-neutrality, the solutions fail at a point around *ξ* = 1 (*x* = ±*R(t*)), where high-energy ions are accelerated most predominantly. The acceleration field *E*_*f*_ (*t*) emerging in the expansion front can be given^26^ by


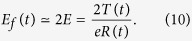


In the following, we use a characteristic time 
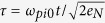
, where 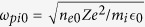 is the initial ion plasma frequency. Here, the electron density is given by the critical density in the relativistic regime:


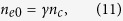


where 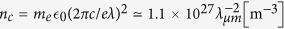 is the non-relativistic critical density. We introduce a normalized acoustic velocity,


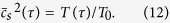


Then, the electric field at the front is given as a function of *τ*:


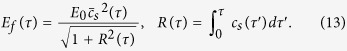


The equation of motion for an ion at the front is written as


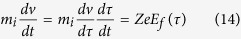


Integrating the equation for *τ*, we obtain the ion velocity


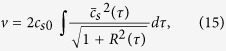


where 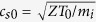. Under the isothermal assumption *T(t*) = *T*_0_ (=const.), the equation is exactly equivalent to 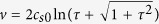, which is the ion velocity reported by Mora^26^. The kinetic energy of an ion is therefore expressed as


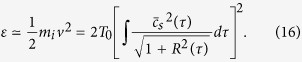


One of the most important parameters governing the ion acceleration is the normalized acoustic velocity 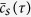, which indicates the enhancement of temperature over the ponderomotive energy. To evaluate 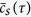, we assume the following function,


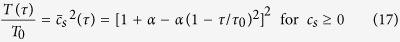


and determine the parameters *α* and *τ*_0_ by fitting the *T(τ*) function to the time evolution of temperature obtained with the 1D PIC simulation shown in Fig. 2. Here, *α* indicates the enhancement of temperature over the ponderomotive energy [*T(τ*) = *T*_0_ for *α* = 0] and *τ*_0_ signifies the time at which the temperature begins to decrease [*T(τ*) = *T*_0_(1 + *α*)^2^ for *τ* = *τ*_0_].

The origin of time (*τ* = 0) is set at the arrival time of the first intensity peak (*t* = 1.5 ps), when *T(τ* = 0) = *T*_0_ = 0.2 MeV, in good agreement with the PIC results. It can be seen from Fig. 2 that the form of Eq. (17) fits well especially for the 1- and 2-pulse cases and the earlier time region (*t* < 6 ps) of the 4-pulse case. For the later times (*t* > 6 ps), since the cooling effect discussed in the previous subsection takes place, a different function might well describe the time dependence of the temperature. If the cooling effect becomes more serious for further longer pulse durations, Eq. (17) should not be used for describing the time dependence of the temperature. To solve the double integral in Eq. (16), we can approximate 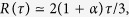 because the average of 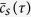 is 2(1 + *α*)/3 over the region of 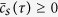.

**Figure 4:**
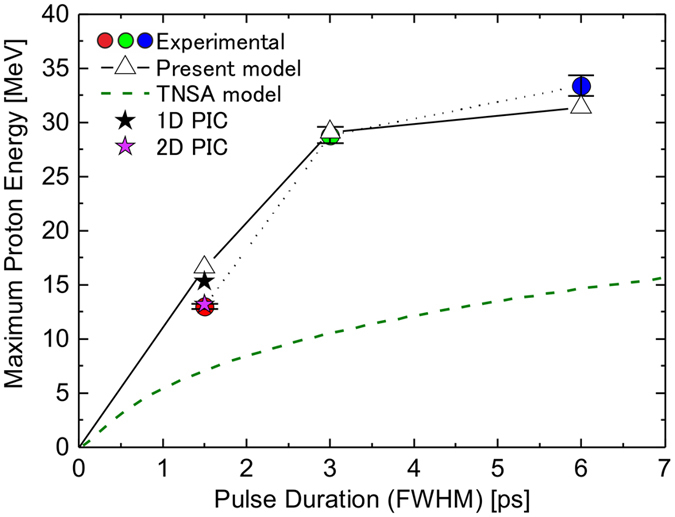
Pulse-duration dependence of proton energies predicted by the present model according to Eq. (16) (triangles), shown with the experimental results (circles) also presented in Fig. 1(a). The results of 1D and 2D PIC simulations (stars) are shown as a comparison. A dashed green curve shows the proton energy predicted by the model of Fuchs *et al*.^25^.

The maximum proton energies derived from Eqs (16) and (17) are compared with the experimental values in Fig. 4, demonstrating a quantitative agreement between them. Here, we set the integration range to be over 0 ≤ *τ* ≤ 2*τ*_0_ on Eq. (16). We emphasize that the proton energies obtained experimentally are explained well by the model involving the electron heating enhanced by the multi-ps laser.

**Figure 5:**
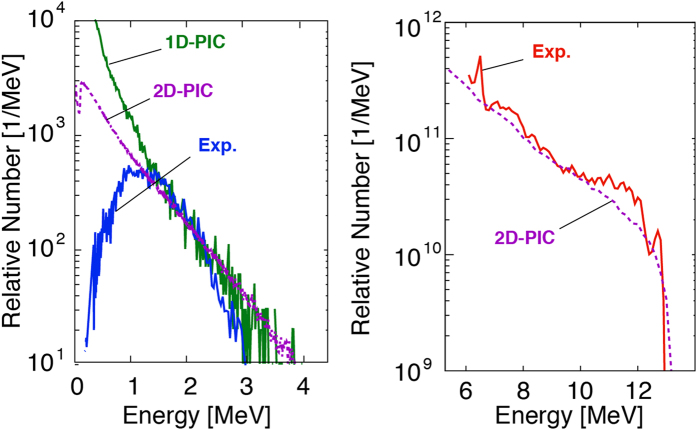
(**a**) The electron energy spectrum for the 1.5-ps duration obtained with 2D PIC simulation assuming a 60 *μ*m focal spot (purple) well agrees to the spectra by the 1D PIC (green) and the experiment (blue) from Fig. 1. (**b**) The proton energy spectra obtained by the 2D PIC (purple) is in well agreement with the experimental result for the 1.5-ps pulse duration.

To demonstrate the applicability of the 1D PIC simulation, we have performed two-dimensional (2D) PIC simulation, PICLS^51^, on proton acceleration assuming the large focal spot, 60 *μ*m (FWHM), as in the experiment. The target is a 5 *μ*m aluminum at solid density with a thin (~10 nm) hydrogen layer placed on the rear surface. The simulation box is 150 *μ*m × 120 *μ*m, which is sufficiently large to avoid the boundary effect. The pulse duration and the intensity are the same with 1D PIC as 1.5 ps (FWHM) and 2.5 × 10^18^ W cm^−2^, respectively. It was found that the hot electron temperature in the energy spectrum obtained in the 2D simulation agrees fairly well to those obtained in the experiment and the 1D simulation as seen in Fig. 5(a). Note the 2D electron spectrum is time-integrated in the same manner with the 1D spectrum. In addition, the maximum proton energy by the 2D PIC (13.2 MeV) agrees well to the experimental result (13 MeV), as shown in Fig. 5(b). The maximum proton energy of 2D PIC is shown also in Fig. 4 with the result of 1D PIC simulation. In the large focal spot used in our experiment and 2D simulation, the plasma expands quasi-one dimensionally. Consequently, the effect of the lateral electron transport is less significant on the sheath formation in the focal spot, compared with that occurs in a smaller focal spot, where the lateral transport is more severe in 3D case than 2D case. Therefore, under the condition of a sufficiently large focal spot, the electron temperature and ion energy obtained in experiments will be in agreement with the results of 2D or 1D simulation even in picoseconds time-scale.

### Conversion efficiency into protons

One of the most important issues to realize a novel ion source by laser is the conversion efficiency (CE) of energy from the laser into the ions. In this study, we have achieved 4.9% CE with a 6-ps, 2.5 × 10^18^-W cm^−2^ (1 kJ) laser pulse.

**Figure 6:**
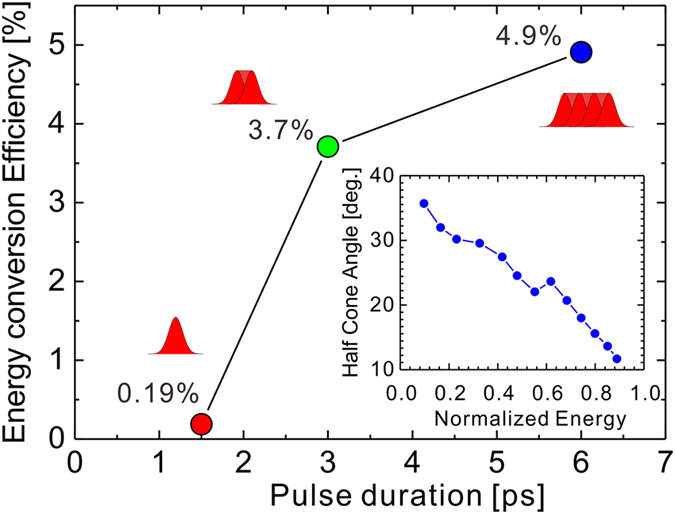
Pulse duration dependence of the conversion efficiency from the laser energy to the protons with kinetic energy above 6 MeV. The inset shows the angular distribution of protons measured for the 1.5-ps, 1.2 × 10^19^-W cm^−2^ case by RCF stack detector as a function of the normalized energy 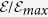.

The beam divergence of protons is measured by a radio-chromatic-film (RCF), HD-V2 stack for the 1.5-ps, 1.2 × 10^19^-W cm^−2^ case, as shown in the inset of Fig. 6, resulting in a cone angle (FWHM) expressed as a function of the proton energy 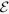 as 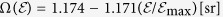, where 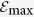 is the maximum proton energy measured. Note this result is consistent with a universal curve in the literature^52^. We assume this proton divergence for 1.5–6 ps duration [Fig. 1(a)] and evaluate the CE into protons above 6 MeV, according to the following formula:


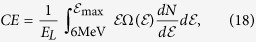


where 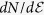 is the proton energy distribution obtained by the TP and *E*_*L*_ is the laser energy in a unit of MeV. As shown in Fig. 6, it is found for the first time that the CE is enhanced by an order of magnitude depending on the pulse duration at a fixed laser intensity and fixed focal spot size. It is noteworthy that the 5% efficiency is achieved with 10^18^-W cm^−2^ intensity. As reported in refs 53 and 54, the large focal spot is one of the reasons explaining the high CE in this study.

## Discussion

In Fig. 4, we demonstrate that our results completely exceed the pulse-duration dependence of proton energies predicted by TNSA model^25^ in a dashed line, which is obtained using our laser parameters. In TNSA model, the electron temperature had been assumed to be constant at the ponderomotive energy, which is clearly lower than the temperature enhanced by the multi-ps laser as seen in this work, resulting in the underestimation of proton energies for the longer pulse duration.

As comparisons with other models, not only TNSA, Passoni *et al*.^55^ theoretically predicted that 30 MeV protons are accelerated with 500 J, 1 × 10^20^ W cm^−2^, when they assumed the electron temperature given by the ponderomotive energy. In our experiment, on the other hand, 29 MeV is achieved with 500 J, 2.3 × 10^18^ W cm^−2^, owing to the multi-ps pulse effect. The difference of two orders of magnitude between the intensities is notable. Zeil *et al*.^56^ analytically formulated the ideal acceleration energy of ions 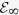, which is realized when the laser pulse duration is infinite: 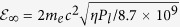, where *P*_*l*_ is the laser power in a unit of Watt and *η* is the energy conversion efficiency from laser into fast electrons. For our laser intensity *I* = 2.3 × 10^18^ W cm^−2^, the efficiency is given by 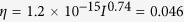
57. The laser power is *P*_*l*_ = 167 TW for our case. Then, the ideal acceleration energy is evaluated to be 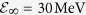. It is interesting to note that our result (33 MeV) is close to the acceleration energy with the infinitely long pulse 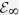.

From the discussions above, we conclude that the multi-ps laser pulse plays a significant role on enhancing the plasma electron temperature, leading to the improvement of accelerated ion energy.

## Methods

### Laser system and diagnostics

Details regarding the LFEX laser system can be found in refs 44 and 45. The ion energy spectra were observed using a TP employing a permanent dipole magnet (0.85 T) and a pair of copper electrodes (12.5 kV/cm). Image plates (IP), BAS-TR2025/Fuji Film, were used as ion detectors^58^ in the TP. A 100-*μ*m thick aluminum foil is placed on the IP to block light and X-rays coming from the laser-plasma interaction chamber. IP signals were converted into proton spectra by using the calibration result in ref. 59, when the proton energy after penetrating the 100-*μ*m aluminum cover was calculated by the PHITS^60^ code.

### Particle-in-Cell simulation

Fully relativistic one-dimensional PIC simulations were performed by the EPIC code^47^. The length of the simulation box is *L*_*x*_ = 81.92 *μ*m or longer in the *x* direction with a mesh size of 10 nm. A *p*-polarized laser field is excited by an antenna located at *x* = 20 nm with a pulse shape defined by 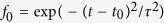 for *t* < *t*_0_, *f*_1_ = 1 for *t*_0_ ≤ *t* < *t*_1_, and 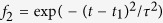 for *t*_1_ ≤ *t* where *τ* = 0.9 ps, the corresponding FWHM is 1.5 ps, and the flat part of the pulse begins at *t*_0_ = 1.5 ps and ends at *t*_1_ = 1.5 ps, 3 ps and 6 ps for the 1-, 2- and 4-pulse cases, respectively. The laser wave length is *λ* = 1.05 *μ*m and the peak normalized amplitude is *a*_0_ = 1.42, which corresponds to an intensity of *I* = 2.5 × 10^18^ W cm^−2^. We set a fully-ionized uniform Al plasma of 5-*μ*m thickness at *x* = 27–32 *μ*m. Note that the electron trajectory and energy in Fig. 4 are obtained in a simulation with *L*_*x*_ = 104.96 *μ*m where the uniform plasma is distributed at *x* = 50–55 *μ*m. On the front side of the target, we put a 1-*μ*m thick preformed plasma, the density of which increases linearly from 0 to the target density. The ion density is *n*_*i*_ = 6.0 × 10^21^ cm^−3^, which is ten times lower than the actual Al solid density, but is still overdense sufficiently for the considered intensity regime *a*_0_~1 with the corresponding electron density *n*_*e*_ = 77.1 *n*_*c*_, where *n*_*c*_ is the cutoff density. The initial temperatures of ions and electrons are *T*_*i*0_ = 0.2 keV and *T*_*e*0_ = 1 keV, respectively. The average numbers of super particles per cell in the distribution region of the plasma, i.e., in the 6 *μ*m length, are 1.7 × 10^2^ and 2.2 × 10^3^ for ions and electrons, respectively. Collisions and ionization processes are not included in the simulations performed in this study.

The PIC simulations including proton acceleration were performed by the PICLS code^51^, which incorporates the Coulomb collisions among charged particles and the dynamic ionizations. The length of the simulation box is 150 *μ*m in the laser propagation direction. In the case of 2D simulation, we used the box 120 *μ*m long in the transverse direction, which is sufficiently larger than the laser focal spot of 60 *μ*m (FWHM). The initial condition of preformed plasma on the target front is same with that used in the EPIC simulation. A thin layer of protons is set on the rear surface of the solid aluminum target with the initial *Z* = 3. The thickness and density of the thin proton layer is ~10 nm and 10 *n*_*c*_, respectively.

## Additional Information

**How to cite this article**: Yogo, A. *et al*. Boosting laser-ion acceleration with multi-picosecond pulses. *Sci. Rep.* **7**, 42451; doi: 10.1038/srep42451 (2017).

**Publisher's note:** Springer Nature remains neutral with regard to jurisdictional claims in published maps and institutional affiliations.
